# Need for Nucleic Acid Testing in Countries with High Prevalence of Transfusion-Transmitted Infections

**DOI:** 10.5402/2012/718671

**Published:** 2012-09-12

**Authors:** Rohit Jain, Pankaj Aggarwal, Gajendra Nath Gupta

**Affiliations:** Department of Pathology and Transfusion Medicine, Santokba Durlabhji Memorial Hospital & Research Institute, Rajasthan, Jaipur 302015, India

## Abstract

*Introduction.* In India, family/replacement donors still provide more than 45% of the collected blood. With increasing voluntary blood donation and the still-prevalent infectious diseases in donors, we need to augment transfusion-transmitted infections (TTIs) testing before use. Our study was aimed to know the seroprevalence of TTIs among the donors of Rajasthan and the need for newer technologies like nucleic acid testing (NAT). *Materials and Methods.* Enhanced chemiluminescence immunoassay (ECi) was used for detection of HBsAg, anti-HIV, and anti-HCV in donor serum. 50% of the blood units which were negative on ECi were randomly selected and subjected to NAT testing for HBV, HCV, and HIV. *Results.* The total seroprevalence of TTIs is 2.62%. Of the randomly selected donor units negative by ECi, 8 turned out to be reactive on NAT testing: 4 were voluntary and 4 were family/replacement donors. Combined NAT yield (NAT reactive/seronegative) for HIV, HCV, and HBV was 0.034% (1 in 2972 donations). All the 8 reactive samples were positive for HBV DNA. *Conclusion.* In countries with a high prevalence of TTIs like India there are likely to be a significant number of window period donations that can be identified by NAT which may be implemented in blood centers allover India with serological testing to provide safe blood and cost alone should not be a deterrent to the government and implementing agencies.

## 1. Introduction

In India, family/replacement donors still provide more than 45% of the collected blood [1]. For a safe blood service in our country, where comprehensive laboratory tests are neither possible nor pragmatic, it is best to switch over to 100% voluntary donations. The key to recruiting and retaining safe blood donors is good epidemiological data on the prevalence (and incidence, where possible) of infectious markers in the general population to identify low-risk donor populations coupled with an effective donor education, motivation and recruitment strategy to recruit new voluntary nonremunerated blood donors from these populations [2]. Voluntary nonremunerated blood donations in India rose from 54.4% in 2006 to 74.1% by January 2010 [3, 4].

 Prevalence is <2% and 0.12–2.5%, respectively [5]. The HIV prevalence rate in the country is 0.29% [3, 4, 6] and HIV prevalence in blood donors is 0.32% [4]. Syphilis prevalence in general population and blood donors is 3.1−51% [4] and 0.85–3% [7], respectively.

With increasing voluntary blood donation and still prevalent infectious diseases in donors, we need to augment transfusion transmitted infection testing before use. Nucleic acid testing (NAT) blood screening for key transfusion-transmitted infections (TTIs) was originally intended to complement serological screening for detection of donations infectious for those viruses. The main advantage of NAT screening is interdiction of new incident cases during the window period infections and identification of occult hepatitis B carrier status which can potentially be infectious [8]. This tool could provide the next large step in improving the safety of blood in India and adding to the epidemiological database of incidence and prevalence of the viral infections [9], where 6 million units of blood are collected annually [4]. Our study was aimed to know the seroprevalence of transfusion-transmitted infections among the donors of Rajasthan, the largest state of Republic of India and the need for newer technologies like NAT testing as there is no previous published data from the region [10].

## 2. Materials and Methods

This retrospective study was conducted at Blood Bank, Santokba Durlabhji Memorial Hospital cum Medical Research Institute, Jaipur, India. It is a state-of-the-art regional blood bank with National Accreditation Board for Hospitals and Healthcare Providers (NABH) accreditation, processing more than 30,000 units per year. A total of 47,558 units of blood were collected from voluntary and HBV prevalence in general population and blood donors in India is 2–8% and 1-2%; HCV replacement donors between January 2009 to September 2010. Voluntary donors had donated blood either in the blood bank or in the camps organized by mobile teams in districts of Rajasthan. There were no voluntary repeat donors. Family/replacement donors had come to the centre to donate blood against that required by patients/relatives. Professional donors are banned as per practice of law. Donors were carefully screened by trained personnel after a complete physical examination and satisfactorily answering the donor's questionnaire. At the end of blood collection, donor samples were obtained for serological/NAT testing. Donor consent was obtained for HIV and other testing.

To determine the prevalence of HBV, HCV, and HIV enhanced chemiluminescence immunoassay (ECi) was used for detection of HBsAg, anti-HIV (anti-HIV 1 and anti-HIV 2), and anti-HCV in donor serum on the VITRO S 3600 Immunodiagnostic System, Ortho Clinical Diagnostics, USA, based on luminescent reaction [11]. To determine the prevalence of syphilis SD bioline syphilis fast 3.0 solid phase, immunochromatographic assay was used for the qualitative detection of antibodies of all isotypes (IgG, IgM, IgA) against *Treponema pallidum* (Anti-TP). Malaria screening was done by trained personnel using Leishman stained peripheral smears on light microscopy. Quality assurance in all tests was maintained as per national standards.

To evaluate the need of adding NAT testing in the blood bank 23,779 (50% of the 47,558) units which were negative on ECi were randomly selected and subjected to NAT testing by Roche Cobas taq screen MPX test on ROCHE COBAS s 201system. It is a qualitative multiplex test that enables the screening and simultaneous detection of HIV-1 groups M and group O RNA, HIV-2 RNA, HCV RNA and HBV DNA (cannot be distinguished) in infected pooled and individual plasma specimen donations followed by specific confirmation by means of automated real-time polymerase-chain-reaction (PCR) on COBAS Taqman analyzer (Roche) with the 1-milliliter extraction method. The test incorporates an internal control for monitoring test performance in each individual test as well as the Amperase enzyme to reduce potential contamination by previously amplified material (amplicon). NAT testing for HBV DNA, HCV RNA, and HIV-1 RNA using the Roche Ampliscreen tests has been accepted by US FDA as confirmatory tests for the serological assays. All the gathered data was evaluated.

## 3. Results

A total of 47,558 donations were received in our blood bank over the period of 21 months of study of which 97.71% were men and 2.29% were women making the donor-sex ratio 42.7 : 1. The numbers of donors in the age range 18–25 (college students), 26–40, and 41–60 were 19269, 21953, and 6336, respectively. 84.65% were voluntary donors and 15.35% were replacement donors. Of these the numbers of HBsAg seropositive donations were 711 (1.5%), HCV seropositive donations were 298 (0.63%), HIV seropositive donations were 159 (0.33%), and anti-TP seropositive donations were 77 (0.16%) making a total of 1245 (2.62%) seropositive donations with voluntary to replacement donor TTI ratio of 1 : 6.8. The prevalence of TTIs in voluntary and replacement donors was 2.53% and 3.11%, respectively. None of the 47,558 donations tested positive for malaria parasite on peripheral smear study (Table 1).

Of the randomly selected 23,779 donor units which were negative by ECi, 8 turned out to be NAT yield (NAT reactive/seronegative) for HIV, HCV, and HBV was 0.034% (1 in 2972 donations). All the 8 reactive samples were positive for HBV DNA and the HBV viral load was ≥12 IU/mL (95% lower limit of detection, 12 IU/mL with 5.82 copies per IU conversion factor) [12]. None was reactive for HIV and HCV.

## 4. Discussion

Disease burden estimations based on sound epidemiological research provide the foundation for public policy. Which diseases and what interventions does public policy need to focus upon are normally derived from such evidence. Accurate estimates of risk of TTIs are essential for monitoring the safety of blood supply and evaluating the efficacy of the currently employed screening procedures. Well-researched, longitudinal data can enable judicious targeting and help decide what needs to be done where, for whom, and when. India has ample evidence of such impacts, often due to the mismatch between disease burden and its causal factors, and the interventions adopted and priorities in resource location [13]. The prevalence of infection among blood donors has been used as a surrogate marker in the population at large. Although certain pitfalls, like the exclusion of people below 18 years and over 60 years and low number of female donors have been cited, it is still an important indicator of the disease burden [5]. The prevalence of TTIs among blood donors in well-structured health care system with a well-organized blood establishment can be used as a reliable tool for statistical estimations of those infectious agents that can be transmitted through blood products and can contribute to statistical estimation of those viruses in the general population [14] (reactive on NAT testing, 4 were voluntary and 4 were family/replacement donors—Combined).

The total seroprevalence of TTIs is 2.62%. Our study supports the rising trend in adult HIV prevalence in Rajasthan [4, 15, 16] at 0.33% which is higher than previous estimates of 0.16% in 2007 [15] and is against the decreasing national trend [3, 4]. HBV and HCV seroprevalence is in concordance with the recently reported 1-2% and 0.12–2.5%, respectively, [5] in blood donors in India and is much higher as compared to that reported in this region by Sood and Malvankar [16] probably because of involvement of hospital-based population from a restricted geographical area in their study. The overall seroprevalence of Syphilis in Rajasthan is 0.16% with a rising trend with age (Table 1) probably due to increase in biological false positive reactions with age [17] or reflecting the increasing promiscuous behavior in men with age.

In India screening for HIV, hepatitis B, and hepatitis C is based on serological testing with recent introduction of NAT testing in few centers. Even after implementing the more sensitive, newest generation of serological tests, a considerable residual risk of transfusion of these viruses' remains [9]. Most populations in resource-limited regions suffer from high prevalence rate of TTIs, and are expected to have more frequent incident cases, as well as more occult carriers. Only countries with a high prevalence and incidence of infection are likely to yield significant number of window period donations. Consequently, NAT screening of TTIs in these populations would be expected to identify more yield cases as compared to the developed world and thus to be more cost effective [8]. In our study of 23,779 cases there were 8 HBV cases and no HIV/HCV window cases were detected on NAT testing. Thus the HBV NAT yield was 1 : 2972 donations which is much higher than in studies done in western Europe and the USA, where the reported prevalence is around 1 : 600,000 to 1 : 350,000 donations [18] and in developing countries like South Africa, Thailand, Kuwait, and Malaysia where the HBV NAT yield was 1 : 52303, 1 : 4868, 1 : 24275, and 1 : 3616 donations, respectively, as compared to high prevalence developing countries like 1 : 232 in Ghana, 1 : 2609 in Egypt, 1 : 501 in Lebanon, 1 : 125 in Iran, 1 : 69 in Mozambique, 1 : 193 in Pakistan, 1 : 865 in Mexico, 1 : 81 in Mongolia, and 1 : 1430 in China [8]. Similarly, with the higher prevalence of HIV-1 and HCV in India compared with the western Europe and the USA (1 : 300,000 to 1 : 3,000,000), it is likely that testing a large number of donors would lead to HCV, and HIV-1 yield cases too [18, 19]. The potential for NAT yield in India is staggering when compared to other countries that have already implemented the technology given the prevalence of these viruses in the population; 5.7 million with the HIV, 12 million with HCV, and 40 million with HBV which represents 10 percent of world's HBV-infected population [9].

Our study shows NAT screening in India would be predicted annually to interdict 336/million (2019 infectious donations of 6 million donations) as compared to 7.5/million (3000 infectious donations of over 400 million donations) in an international survey that included 37 countries that reported results over a 10 year long period extending from 1998 to 2008 that would have been missed by serological screening methods [20]. Similarly hepatitis B virus nucleic acid testing in Chinese blood donors would detect an additional 9964 viremic donations per year [21]. China and India are two countries with large populations where the adoption of NAT could have a significant impact on the rate of TTIs. Around the world, more than 53 million units of blood are screened with NAT annually. 100% of the USA blood supply is screened with NAT for HIV-1, HCV, HBV, and West Nile virus [22]. Although the yield of NAT-only units is modest relative to the yield of serological screening, the infectivity of viremic donations detected by NAT (with or without detectable serological markers) is very high. Hence, the relative impact of NAT screening is arguably greater than that of serological screening, although the existence of seropositive but NAT-negative donations indicates that serological screening must be maintained even with the most sensitive NAT testing performed on individual donations. Consequently the incremental cost-effectiveness of NAT is marginal since the safety benefits used in these calculations are restricted to the prevention of transmission of NAT-only yields and the cost of NAT testing remains relatively expensive [19]. Recent technical advances might be able to address these limitations [23, 24].

Family donors are not safe as voluntary donors and voluntary nonremunerated repeat blood donors are perceived to be safer than the first time blood donors [1, 25]. In our study there were no repeat voluntary donors and even after increasing first time voluntary blood donation (approximately 85%) it is noted that NAT needs to be added to the serological testing as we had an equal number (4 each) of NAT positive cases in voluntary and family/replacement donors [9, 18, 26, 27]. We may follow the example of South Africa with the highest prevalence of HIV in the general population (18%) who has implemented 100% voluntary donation and >80% repeat donations leading to the decrease in the prevalence of HIV in the donor population by 100-fold which is far lower than the other countries that have lower general population prevalence. Moreover, South Africa has decided that all blood donations will be screened using molecular-based assay that detect the nucleic acids of HIV, HBV, and HCV viruses in a multiplexed individual donation format in parallel with serological assays [28]. Pooling strategies integrated with antibody testing would be cost-effective for laboratories with large testing volumes compared to individual testing [29–33]. In their families, beyond the direct costs of medications, monitoring, and medical care, additional costs include the long-term lost earning of transfusion-transmitted infected individuals as well as of their household members who also provide care. A clearer understanding of the financial burden of healthcare for transfusion-transmitted infected Indians can allow policy makers and planners to better allocate limited resources [34].

## 5. Conclusion

The general public may be idealistic in their belief that risk-free blood products are achievable in today's world. In countries with a high incidence of infection like India there are likely to be significant number of window period donations that can be identified by NAT as in our study. The actual benefit has first to be determined and should be balanced against the complexity and high cost of performing NAT, including the infrastructure required. NAT may be implemented in blood centers all over India with serological testing to provide safe blood and cost alone should not be a deterrent to the government and implementing agencies.

## Figures and Tables

**Table 1:** Prevalence of HBsAg, HCV, HIV, and anti-TP in blood donors of Rajasthan, India (voluntary donors = 40,257 replacement donors = 7,301, total donors = 47,558).

	HBsAg	HCV	HIV	Anti TP	Total
Overall prevalence (%)	1.5	0.63	0.33	0.16	2.62
Men : women	100.6 : 1	58.6 : 1	30.8 : 1	24.7 : 1	53.7 : 1
Seropositivity ratio					
Prevalence in voluntary donors (%)	1.45	0.61	0.32	0.15	2.53
Prevalence in Replacement donors (%)	1.77	0.71	0.41	0.22	3.11
Age-specific prevalence					
18–25 (%)	1.08	0.60	0.35	0.10	2.13
26–40 (%)	1.8	0.63	0.31	0.14	2.88
41–60 (%)	1.7	0.74	0.35	0.43	3.22
